# Challenges and strategies: treating spontaneous pneumothorax in massive pulmonary langerhans cell histiocytosis in children

**DOI:** 10.1590/1984-0462/2025/43/2024076

**Published:** 2024-11-29

**Authors:** Letícia Helena Kaça do Carmo, Luiz Augusto Marin Jaca, Luiz Miguel Vicente Barreiros, Gabriela Marengone Altizani, Leticia Fontanini, Maristella Bergamo Francisco dos Reis, Mauricio André Pereira da Silva, Marcel Koenigkam Santos, Monica Cypriano, Ygor Aluísio Moura, Henrique Lederman, Carlos Alberto Scrideli, Elvis Terci Valera

**Affiliations:** a Universidade de São Paulo, Faculdade de Medicina de Ribeirão Preto, Ribeirão Preto, SP, Brazil.; b Univeridade Federal de São Paulo, Instituto de Oncologia Pediátrica, Grupo de Apoio ao Adolescente e à Criança com Câncer, São Paulo, SP, Brazil.

**Keywords:** Langerhans-cell histiocytosis, Lung, Video-assisted thoracic surgery, Case report, Histiocitose de células de Langerhans, Pulmão, Cirurgia torácica videoassistida, Relato de caso

## Abstract

**Objective:**

The objective of this study was to report two cases of successive multiple spontaneous bilateral pneumothorax in children with massive lung involvement due to Langerhans cell histiocytosis (LCH), emphasizing the possibility of this differential diagnosis for the general pediatrician. Additionally, published cases describing pediatric patients with pulmonary manifestations of LCH were reviewed in the literature.

**Case description:**

Case #1: A 3-year-old male patient with a sudden episode of spontaneous right-sided pneumothorax, surgically drained. After 2 months, he experienced two new episodes of contralateral pneumothorax. A pulmonary lymph node biopsy revealed the diagnosis of LCH. He underwent bilateral video-assisted thoracic surgery and mini-thoracotomy with mechanical pleurodesis, in addition to chemotherapy, requiring prolonged hospitalization. Case #2: A 4-year-old boy with progressive dyspnea and wheezing for 5 months. A pulmonary biopsy revealed LCH. He developed significant respiratory distress and right pneumothorax, requiring drainage. Silver nitrate pleurodesis and different chemotherapy regimens were performed. Both patients responded well to multiple chemotherapy treatments, surgeries, and intensive care support.

**Comments:**

LCH is a challenging disease. Its clinical manifestation is variable, and pulmonary involvement occurs in about 10–15% of cases. We consider specialized surgical management and multidisciplinary support essential for the treatment of patients with massive pulmonary LCH. Although rare, massive pulmonary involvement by LCH should be considered in cases of recurrent pneumothorax in children.

## RESUMO

**Objetivo:**

Relatar dois casos de pneumotórax bilateral espontâneo múltiplo sucessivo em crianças com envolvimento pulmonar maciço por histiocitose de células de Langerhans (HCL), alertando para a possibilidade desse diagnóstico diferencial para o pediatra geral. Ademais, casos publicados que descrevem pacientes pediátricos com manifestações pulmonares de HCL foram revisados na literatura.

**Descrição do caso:**

Caso 1: Paciente masculino, três anos, com episódio súbito pneumotórax espontâneo à direita, drenado cirurgicamente. Após dois meses, apresentou dois novos episódios de pneumotórax contralateral. Biópsia linfonodal pulmonar revelou diagnóstico de HCL. Foi submetido a cirurgia torácica assistida por vídeo (VATS) bilateral e minitoracotomia com pleurodese mecânica, além de tratamento quimioterápico, necessitando de internação prolongada. Caso 2: Menino, quatro anos, apresentando dispneia progressiva e sibilância por cinco meses. Biópsia pulmonar revelou HCL. Evoluiu com importante desconforto respiratório e pneumotórax direito, necessitando drenagem. Foi realizada pleurodese com nitrato de prata e diferentes esquemas de quimioterapia. Ambos os pacientes evoluíram bem após tratamentos quimioterápicos múltiplos, cirurgias e suporte intensivo.

**Comentários:**

A HCL é uma doença desafiadora. Sua manifestação clínica é variável, e o envolvimento pulmonar ocorre em cerca de 10–15% dos casos. Consideram-se o manejo cirúrgico especializado e o suporte multidisciplinar essenciais para o tratamento de pacientes com HCL pulmonar maciça. Ainda que raro, o envolvimento pulmonar maciço por HCL deve ser considerado em quadros de pneumotórax recorrentes em crianças.

## INTRODUCTION

Histiocytosis is a challenging disease group, presenting either as localized cases with excellent survival or complex forms with poorer prognostics. This group of diseases is characterized by the expansion of different cell lineage spectrums such as dendritic cells (DC3), histiocytes, and monocyte/macrophages.^1^ Currently, histiocytosis is categorized into specific subgroups: Langerhans-related (L), cutaneous and mucocutaneous histiocytosis (C), malignant histiocytosis (M), Rosai-Dorfman disease (R), and hemophagocytic lymphohistiocytosis/macrophage activation syndrome (H). Langerhans cell histiocytosis (LCH) is the most common subtype, characterized by the expansion of CD1a+/CD207+ myeloid dendritic cells in different tissues and organs.^2^ LCH in children and adolescents may rarely be asymptomatic or oligosymptomatic. The primary manifestations of LCH commonly include painful bone lesions and cutaneous rash. Often, nonspecific symptoms such as fever, diminished appetite, weight loss, fatigue, irritability, and behavioral changes may become prominent. Eventually, more distinctive symptoms related to the involvement of bones, skin, the pituitary gland, liver, spleen, the hematopoietic system, lungs, lymph nodes, the central nervous system, thymus, and the gastrointestinal tract may arise.^3^ The most affected sites in children are bones (injured in 80% of patients), skin, and the pituitary gland.^4^ Due to the variable spectrum clinical presentation of LCH histiocytosis in the pediatric age, diagnosis can be challenging. The diagnosis of LCH relies on clinical and radiological observations along with histopathological examinations revealing tissue infiltration by histiocytes displaying ultrastructural or immunophenotypic characteristics (particularly CD1a and S100 positivity) of Langerhans cells. Pulmonary involvement in histiocytosis occurs in about 10–15% of all LCH cases and is more often observed in the adult setting, particularly related to smoking habits.^1,4^ Massive pulmonary involvement of LCH (PLCH) is exceedingly rare, particularly in the pediatric population.

Here, two pediatric cases involving multiple and successive spontaneous bilateral pneumothoraces in children with PLCH are presented. Challenges associated with determining the most effective treatment approaches and providing optimal surgical support for these challenging cases are discussed. Additionally, aggregated data from published cases describing surgical interventions in children with PLCH were collected. By detailing these two uncommon pediatric cases, the objective is to draw attention to the possibility of PLCH in the differential diagnosis of spontaneous pneumothorax in children and adolescents.

## CASE REPORT

**Figure 1:**
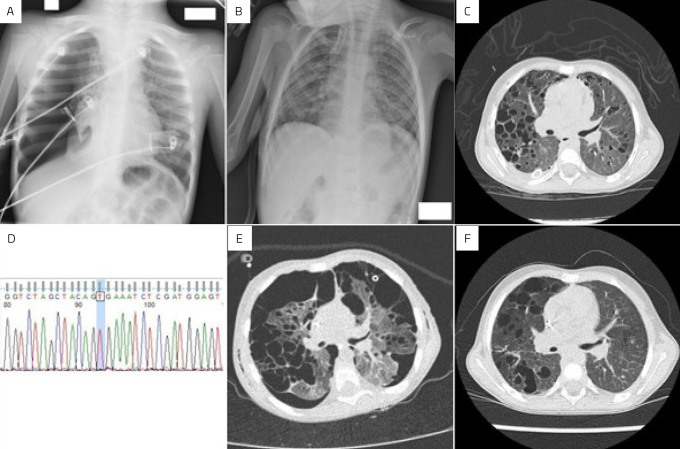
(A) X-ray of admission showed a right pneumothorax. (B) X-ray after thoracostomy, showing pulmonary expansion and diffuse interstitial lung disease. (C) CT after thoracostomy, showing diffuse cystic lung disease. (D) Electropherogram showing the absence of mutation (blue column) at codon 600 of the BRAF gene (wild-type). (E) CT at the beginning of remission therapy, during new bilateral pneumothorax. (F) CT at the end of maintenance therapy shows good pulmonary expansion and reduced number and size of pulmonary cysts.

A 3-year-old male presented with sudden dyspnea and chest pain on the day of admission. At the admission to the emergency room, he presented with tachycardia, weak peripheral pulses, prolonged capillary refill time, and diminished breath sounds at the right hemithorax. A chest X-ray depicted a spontaneous pneumothorax (Figure 1A). The pediatric surgery team placed a tube thoracostomy with satisfactory expansion of the right lung (Figure 1B). Computed tomography (CT) showed diffuse cystic disease in both lungs (Figure 1C), with cervical and mediastinal enlarged lymph nodes. A chest tube was removed after 4 days, and he was discharged to consult a pediatric pulmonologist. Serologies for HIV, cytomegalovirus, and fungi (paracoccidioidomycosis and histoplasmosis) were all negative. Bone X-rays found one small (5.6 mm) osteolytic lesion on the diaphysis of the proximal femur. Bone scintigraphy, abdominal ultrasound, bone marrow aspirate and biopsy, and pituitary function were also normal. Lung and cervical lymph node biopsies confirmed the diagnosis of LCH (CD1a, CD68, CD163, and S100 positive). *BRAF*V600E mutation was negative in the specimen (by PCR/SANGER method) (Figure 1D).

The patient started treatment with LCH-IV 2015 protocol^5^ (vinblastine/prednisone). Despite treatment, he had multiple episodes of clinical deterioration, hypoxia, and hypertensive pneumothoraxes, requiring multiple (22) chest drainages (Figure 1E) with several admissions to the pediatric intensive care unit (PICU). The surgical team decided to perform a bilateral video-assisted thoracic surgery (VATS) in two stages to release the pleural adhesions, making a single pleural cavity, and optimize the drainage, in addition to mechanical pleurodesis. The patient received intense and daily physical therapy treatment. LCH therapy was switched to cladribine; after three cycles of the new chemotherapy, there was evident clinical improvement (no need for oxygen supplementation). Subsequently, he underwent a right mini-thoracotomy with mechanical pleurodesis and a lung biopsy that showed no residual tumor. The patient started maintenance with mercaptopurine/methotrexate/vinblastine/prednisone. He is currently at the end of maintenance, asymptomatic, with no limitations on his daily activities. There was a significant improvement in pulmonary CT (Figure 1F).

**Figure 2:**
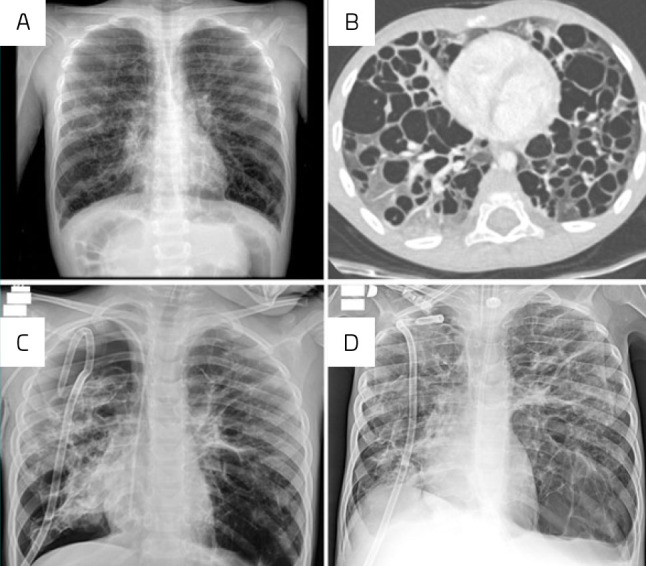
(A) An initial chest X-ray led to the diagnosis of pneumonia. (B) Chest CT showed multiple and disseminated pulmonary cysts throughout the lung parenchyma. (C) Chest X-ray after draining the right hemithorax. (D) A new chest X-ray depicting enlargement of the bubble on the left base, with mediastinal deviation to the right side.

The second case (a 4-year-old previously healthy male) started with progressive dyspnea and wheezing 5 months before admission. The mother is healthy, while the father had a history of undergoing three thoracic drainage surgeries due to “lung bubbles,” although the specifics were not defined, and he is not undergoing any follow-up. The child was initially diagnosed with pneumonia based on a chest X-ray (Figure 2A) and received amoxicillin for 7 days, without improvement. Due to the persistent symptoms, he was hospitalized for a diagnostic investigation of bronchospasm. A chest CT scan was performed (Figure 2B), and he was treated with ceftriaxone, bronchodilators, and oxygen support. After 8 days, he was discharged and referred for outpatient follow-up with a pulmonologist. Upon evaluation by a pulmonologist, the patient presented with tachypnea, significant respiratory effort, and subdiaphragmatic, intercostal, and sternal retractions. He was admitted for a diagnostic workup. Among other assessment tests, a lung biopsy was performed, revealing PLCH with CD1a positivity and a negative *BRAF*V600E mutation. Staging workups (serologies, complete blood count, biochemistry, skeletal radiography, bone scintigraphy, and echocardiogram) showed no evidence of involvement of other organs or systems. In preparation for chemotherapy, he developed significant respiratory distress and auscultation consistent with right-sided pneumothorax, requiring orotracheal intubation and drainage of the right hemithorax (Figure 2C). He began treatment with vinblastine/prednisone, responding initially well, despite several complications (subcutaneous emphysema, KDIGO stage-3 acute kidney injury, ventricular extrasystoles, fever, neutropenia, urinary infection with *Candida metapsilosi*s, and positive blood culture for *Streptococcus mitis*). Yet, due to subsequent deterioration of lung function and the need for reintubation, the protocol was switched to cytarabine (Ara-C) 100 mg/m^2^/day×5 days, and pleurodesis with silver nitrate was performed. While receiving the third cycle of Ara-C, a new episode of pneumothorax was depicted (Figure 2D), leading to the insertion of a second drain into the left hemithorax, followed by another episode, leading to the placement of a third chest drain. Due to prolonged intubation, a tracheostomy was performed, followed by bilevel-positive airway pressure ventilation. The chemotherapy regimen was again modified to the Japanese protocol arm B (Japan Langerhans Cell Histiocytosis Study Group-96)^6^ with gradual clinical and radiological improvement. The drains were successively removed, and after 167 days of hospitalization, including 143 days in the PICU, the patient was discharged home. The child, now 6.5 years of age and off therapy for 1 year, is well and thriving; a few minor complications since treatment completion were seen, including a new pneumothorax, which was successfully resolved with drainage and pleurodesis.

## DISCUSSION

Histiocytosis constitutes a heterogeneous group of disorders, currently assumed to be of neoplastic nature, caused by tissue infiltration of cells with histological characteristics shared with macrophages or dendritic cells.^2^ The pathogenesis of this condition involves signaling pathway mutations, especially in the mitogen-activated protein kinase and BRAF pathways — both frequently associated with other neoplastic origins.^7^ Indeed, the presence of a somatic BRAFV600E mutation can be used to confirm an LCH diagnosis,^8^ and targeted therapy with anti-BRAF drugs has shown favorable clinical results in LCH patients.^9^ Those disorders are classified according to the WHO classification of lymphoid neoplasms,^10^ or according to the Histiocyte Society classification, which divides the disorders into five categories according to molecular, histologic, and clinical features.^1^ Clinical presentation, characteristic imaging findings, and histopathological analysis — through a biopsy fixed in buffered formalin — provide the definitive diagnosis.^1^

Due to the unique nature of the lung involvement in PLCH in these non-smoking preschoolers, a literature review of the PubMed database was conducted to identify similar cases. Multiple combinations of the uniterms “LCH,” “children,” “pulmonary,” and “case report” were applied (02/2024). Publications describing children with PLCH requiring surgical intervention were selected for a full review, and additional cases were searched in the references of the selected studies. A total of 12 case reports, detailing 13 patients like the two cases presented here, were reviewed (Table 1)^11-22^. Most of the patients were male, of varying ages, all presenting with spontaneous pneumothorax. Interestingly, in two of the cases, patients were adolescents who smoked^12,18^. The most common intervention was chemical pleurodesis, frequently performed through VATS^12,15,19^. VATS was also used in two cases for lung biopsy^12,17^. Four patients presented progressive disease and died during treatment^13,20-22^. The two adolescents who smoke agreed on smoking cessation, reporting no symptoms on follow-up^12,18^. The remaining patients started on chemotherapy and had no recurrence of pneumothorax.

**Table 1:** Summary of the reported cases of Langerhans cell histiocytosis causing primary spontaneous pneumothorax treated with surgical intervention.

Reference	Year	Age	Sex	LCH type	Pneumothorax description	Surgical intervention
Aziz et al.^11^	2011	9 years	Male	MLCH	SBRP	Bilateral thoracotomy, pleurectomy, and pleural abrasion
Addams et al.^12^	2007	17 years	Male	IPLCH	SBRP	VATS with lung biopsy and pleurodesis
Alavi et al.^13^	2007	2 months	Female	MLCH	SBRP	Chemical pleurodesis through tube thoracostomy
Benattia et al.^14^	2021	7 years	Male	IPLCH	Left-side pneumothorax	Surgical pleurodesis
Betansos et al.^15^	2023	10 years	Male	MLCH	SBRP	VATS, blebectomy, and mechanical and chemical pleurodesis
Joshua et al.^16^	2021	1 year	Male	MLCH	Spontaneous recurrent right-sided pneumothoraces	Thoracoscopic, partial decortication, decompression of bullae, lung biopsy, chemical pleurodesis through a chest tube, thoracotomy, and open decortication with parietal pleurectomy and bullae excision
Márquez-Vega et al.^17^	2011	6 years	Male	IPLCH	Left tension pneumothorax	VATS with lung biopsy and bronchoalveolar lavage
Onorato et al.^18^	2022	16 years	Male	MLCH	SBRP	VATS with right apical lung biopsy
Rajvanshi et al.^19^	2021	18 months	Male	IPLCH	Three episodes of spontaneous unilateral pneumothorax	VATS with deroofing of the largest cyst and chemical pleurodesis
Soyer et al.^20^	2019	3 years	Male	MLCH	SBRP	Chemical pleurodesis through tube thoracostomy, thoracoscopic bullae excision, and pleural decortication
Varkki et al.^21^	2013	6 years	Male	IPLCH	SBRP	Thoracoscopic lung biopsy and physical and chemical left pleurodesis
Yule et al.^22^	1997	Patient A: 16 months Patient B: 13 years	Both Male	Both MLCH	Both SBRP	Patient A: chemical pleurodesis through tube thoracostomy Patient B: VATS and bilateral pleurectomy through a median sternotomy

In our search on PubMed, 12 case reports, containing 13 patients, were selected for this review. LCH: Langerhans cell histiocytosis; MLCH: multisystem Langerhans cell histiocytosis; IPLCH: isolated pulmonary Langerhans cell histiocytosis; SBRP: spontaneous bilateral recurrent pneumothorax; VATS: video-assisted thoracic surgery.

Spontaneous pneumothorax is a relatively rare condition, although its overall incidence is increasing, especially among adolescents and young adults^23^. The most common causes of spontaneous pneumothorax are emphysematous bleb, asthma, and tobacco use^24^. There is no specific guideline for managing this condition in children to date^25^, underscoring the importance of early etiological diagnosis. Although uncommon, the possibility of PLCH must be included in the differential diagnosis, especially in the numerous recurrences of the pneumothorax. The clinical cases described herein, along with literature review data, emphasize the role of surgical intervention in managing recurrent pneumothoraxes in PLCH, along with chemotherapy and an experienced multidisciplinary team to treat these patients.

## Data Availability

The authors confirm that the data supporting the findings of this study are available within the article. Additional data may be made available upon reasonable request and approval by the ethical committee. This study was approved by the National Research Ethics Committee: 74550923.2.0000.5440.
